# IL-4 Attenuates Th1-Associated Chemokine Expression and Th1 Trafficking to Inflamed Tissues and Limits Pathogen Clearance

**DOI:** 10.1371/journal.pone.0071949

**Published:** 2013-08-26

**Authors:** Christopher A. Lazarski, Jill Ford, Shoshana D. Katzman, Alexander F. Rosenberg, Deborah J. Fowell

**Affiliations:** 1 David H. Smith Center for Vaccine Biology and Immunology, Aab Institute of Biomedical Sciences, Department of Microbiology and Immunology, University of Rochester, Rochester, New York, United States of America; 2 Division of Allergy, Immunology and Rheumatology, University of Rochester Medical Center, Rochester, New York, United States of America; Louisiana State University, United States of America

## Abstract

Interleukin 4 (IL-4) plays a central role in the orchestration of Type 2 immunity. During T cell activation in the lymph node, IL-4 promotes Th2 differentiation and inhibits Th1 generation. In the inflamed tissue, IL-4 signals promote innate and adaptive Type-2 immune recruitment and effector function, positively amplifying the local Th2 response. In this study, we identify an additional negative regulatory role for IL-4 in limiting the recruitment of Th1 cells to inflamed tissues. To test IL-4 effects on inflammation subsequent to Th2 differentiation, we transiently blocked IL-4 during ongoing dermal inflammation (using anti-IL-4 mAb) and analyzed changes in gene expression. Neutralization of IL-4 led to the upregulation of a number of genes linked to Th1 trafficking, including CXCR3 chemokines, CCL5 and CCR5 and an associated increase in IFNγ, Tbet and TNFα genes. These gene expression changes correlated with increased numbers of IFNγ-producing CD4+ T cells in the inflamed dermis. Moreover, using an adoptive transfer approach to directly test the role of IL-4 in T cell trafficking to the inflamed tissues, we found IL-4 neutralization led to an early increase in Th1 cell recruitment to the inflamed dermis. These data support a model whereby IL-4 dampens Th1-chemokines at the site of inflammation limiting Th1 recruitment. To determine biological significance, we infected mice with *Leishmania major*, as pathogen clearance is highly dependent on IFNγ-producing CD4+ T cells at the infection site. Short-term IL-4 blockade in established *L. major* infection led to a significant increase in the number of IFNγ-producing CD4+ T cells in the infected ear dermis, with no change in the draining LN. Increased lymphocyte influx into the infected tissue correlated with a significant decrease in parasite number. Thus, independent of IL-4's role in the generation of immune effectors, IL-4 attenuates lymphocyte recruitment to the inflamed/infected dermis and limits pathogen clearance.

## Introduction

IL-4 plays a key role in immune responses to parasitic helminths and allergic inflammation associated with atopic disease. IL-4 deficient mice show a marked delay in the clearance of helminth infection and over-expression of IL-4 drives local allergic inflammation. It has become clear that many cell types can produce IL-4 including activated T cells, mast cells, basophils and eosinophils. The importance of IL-4 in these responses comes from its ability to drive B cell isotype switch to IgE, to support the differentiation and maintenance of Th2 effectors and to coordinate the accumulation of Type 2 immune effectors in target tissues [1], [2], [3].

In addition to an IL-4 positive feedback loop for Type 2 responses [1], IL-4 also acts as a negative regulator of Th1 and Th17 inflammation. During Th differentiation, IL-4 signaling inhibits the differentiation of naïve CD4+ T cells into Th1 or Th17 effectors [4], [5], [6]. IL-4 antagonizes Th1 differentiation by repressing IL-12 signaling through inhibition of IL-12Rβ2 expression [7] or STAT4 [8]. Additionally, IL-4/STAT6 can negatively regulate Th1s by driving repressive epigenetic modifications [9], [10] and by GATA3-dependent blockade of Runx3-dependent IFNγ gene expression [11], [12]. IL-4 mediated inhibition of Th17s is less well characterized but may be controlled by IL-4 induced expression of Gfi-1 that antagonizes TGFβ driven Th17 differentiation [13]. IL-4 also controls inflammation by regulating the balance between pro-inflammatory classically activated, or M1, macrophages and alternatively activated (AAM), or M2, macrophages [14], [15], [16]. While IL-4 was first described to inhibit macrophage activation and suppress TNF and IL-6 production it is now clear that IL-4 can also positively induce ‘alternative’ macrophage functions associated with chronic infection, allergic inflammation and tissue fibrosis [17], [18], [19].

The balance of immune effectors in infected or inflamed tissues is also controlled by the local differential recruitment of innate and adaptive cell types [20], [21]. Th1, Th2 and Th17 effector cells preferentially express distinct patterns of chemokine receptors that may promote recruitment to discrete types of inflammation in tissues. Positive feedback loops involving cytokines and chemokines appear to amplify and polarize tissue inflammation. GATA3 directs Th2 differentiation and induces the expression of CCR4 [22] while IL-4 activates STAT6 signaling to induce the upregulation of CCR4 ligands, CCL17 and CCL22 [23]. Indeed, IL-4 produced by innate immune cells and STAT6 signaling in non-hematopoietic cells are critical for the recruitment of Th2 cells and other Type 2 innate effectors to the target tissue for pathogen clearance and allergic inflammation [24], [25]. Similar coupled expression between Tbet and CXCR3 and IFNγ and induction of CXCR3 ligands in tissues occurs in Th1 dominated responses [26], [27]. The role of IL-4 in negatively regulating Th1 recruitment has been less well studied.

Our previous studies in mice infected with *Leishmania major* revealed that early in infection, the infected dermis contained an IL-4-dominant immune infiltrate that was in contrast to the generation of a mixed IL-4 and IFNγ anti-*Leishmania* response in the draining lymph node [28]. Notably, *L. major* infection correlated with a down-regulation of CXCR3 chemokine expression in the in vivo infected dermis. *Leishmania* induction of the Type 2 inflammation-associated chemokine CCL7 (MCP3) partially accounted for the dominant IL-4 response but had no role in limiting the number of IFNγ producers in the infected dermis [28]. Thus the factors that limited the accumulation of IFNγ-producers at the infection site had not been identified. Here, using short-term depletion of IL-4, we identify a group of genes associated with Th1 recruitment that are negatively regulated by IL-4 in inflamed tissues. IL-4 neutralization led to the rapid accumulation of IFNγ-producing Th1 cells into the inflamed tissue. The increased number of IFNγ-producers in the tissue following IL-4 blockade was not due to increases in the generation of new IFNγ-producers in draining lymph node but was associated with enhanced Th1 cell recruitment to the target tissue. Thus, IL-4 production in infected or inflamed tissues amplifies local Th2 immunity in part through negatively regulating Th1 recruitment. A number of pathogens such as *Francisella tularensis*, *Mycobacterium tuberculosis*, *Toxoplasma gondii* and *Leishmania* species may exploit IL-4's regulatory effects in the local tissue to limit recruitment of host anti-microbial Th1 responses [29], [30], [31], [32]. In the context of *L. major* infection, short-term IL-4 blockade did indeed facilitate an increased recruitment of IFNγ-producing effector cells into the infected dermis and boosted parasite clearance.

## Materials and Methods

### Mice

BALB/c mice were purchased from either the National Cancer Institute or the Jackson Laboratory. DO11.10 TCR transgenic mice crossed with the IL-4 reporter (4get) [33] were bred at the University of Rochester. Mice were age/sex matched and 6–8 weeks old. All mice were maintained in the pathogen-free animal facility at the University of Rochester School of Medicine and Dentistry, Rochester, NY. All animal experimentation was conducted in accordance with the recommendations of the Weatherall report and followed the guidelines of the University of Rochester's University Committee on Animal Resources (UCAR), the Institutional Animal Care and Use Committee (IACUC). All animal experimentation in this study was reviewed and approved by the University of Rochester's University Committee on Animal Resources (UCAR), the Institutional Animal Care and Use Committee (IACUC).

### ELISPOT

Individual ears were incubated in 1 mL of 1 mg/mL collagenase/dispase (Roche) containing 1 mM CaCl_2_ for 30′ with agitation at 37°. After 30′, EDTA was added to a final concentration of 5 mM and ears were returned to 37° for another 5′ to complete dissociation. Ears were gently pushed through a strainer and washed twice with HBSS/2% FCS. Cell suspensions were spun down and re-suspended in 200 µL of RPMI/10% FCS per ear. Draining lymph nodes (cervical) were gently pushed through a strainer and cells isolated and counted, adjusting concentrations to 1×10^6^ per 100 µL. Ear titers began at 0.5 ear equivalents per well (100 µL) while lymph node titers began at 1×10^6^ cells per well (100 µL), and both were diluted 1∶1 in RPMI/10% FCS in elispot plates (Millipore) that had been pre-coated with anti-IL-4 or anti-IFNγ. LN and ear cells were re-stimulated with either 1 µM OVA peptide [323–339] (for cells from OVA/CFA immunized mice) or soluble *Leishmania* antigen (SLA) (equivalent to 1×10^6^ parasites/ml) (for cells from *L. major* infected mice) or media alone overnight and plates were developed as previously described [28], [34]. Plates were scanned using an ImmunoSpot reader (Cellular Technology Limited) and spots were counted and analyzed using CellSpot software v3.0.

### FACS staining

Conjugated antibodies were purchased from either ebiosciences or Biolegend. Cell suspensions were incubated with unlabeled anti-CD16/32 (clone 2.4G2) to block Fc receptors. For detection of cell transfers, cells were stained with α-Thy1.1 FITC, α-Thy1.2 PE, α-CD4 PerCP-Cy5.5, α-CD3ε Alexa Fluor 700, α-CD45 Pacific Blue, LIVE/DEAD Fixable Near-IR (Life Technologies), α-CD8a APC-Cy7, α-CD11b APC-Cy7, and α-CD19 APC-Cy7. Cells were analyzed on an LSR II (BD Biosciences). Percent and number of live, CD45+, CD4+, Thy1.1−, and Thy1.2+ cells (and negatively gated for CD8alpha, CD11b and CD19 expression), from the ear were analyzed and counted via FlowJo software (TreeStar). For innate and adaptive immune cell phenotyping, cells from the ear were stained with α-CD45 FITC, α-Gr-1 (cone RB6-8C5) Pacific Blue, α-B220 Pacific Orange, α-CD4 PE-Cy5, α-CCR3 PerCP-Cy5.5 or α-CCR3 Alexa Fluor 647, α-DX5 PE-Cy7, α-FcεRIα-Biotin+Streptavidin-QDot 605, α-CD11b Alexa Fluor 700, α-CD11c PE-Texas Red, and LIVE/DEAD Fixable-Near IR. Live, CD45+, CD4−, B220−, Gr-1−, FceRIα+, SSC low, FSC low cells were identified as basophils. Live, CD45+, CD4−, B220−, Gr-1 high, FceRIα−, CD11b+, SSC intermediate, FSC low cells were identified as neutrophils. Live, CD45+, CD4−, B220−, CCR3+, Gr-1 intermediate, FcεRIα−, CD11b low, SSC high, FSC intermediate cells were identified as eosinophils.

### Immunizations and infections

BALB/c mice were immunized in ear dermis with 10 µL of 1 mg/mL OVA protein (Sigma-Aldrich) emulsified in complete Freund's adjuvant (CFA) (Sigma) (10 µg total OVA protein per ear) or 10 µL of PBS/CFA alone using 29.5 gauge insulin syringe (Beckton Dickinson). BALB/c mice were infected in ear dermis with 2×10^5^ infectious *Leishmania major* promastigotes in 10 µl PBS as previously described [28]. Parasite titres were determined by limiting dilution analysis. Briefly, ear suspensions were resuspended in 450 µl cRPMI. 50 µL of the suspension was plated in a 96 well flat bottom plate containing 150 µL of the parasite growth medium, HOSMEM II. 10 fold dilutions were carried out across the wells of the plate and titers were determined at 6 and 10 days post plating. For IL-4 neutralizing experiments, mice were immunized with CFA/OVA or infected with *Leishmania major* as described above. On days 7 and 10 after immunization/infection, mice received 1 mg 11B11 antibody or 200 µl 1× PBS i.p. Ears and draining (cervical) lymph nodes were harvested on day 14, four days after the last 11B11 or PBS injection.

### *In vitro* CD4+ Th1 cell cultures and Adoptive Transfers

CD4+ T cells were enriched from pooled lymph nodes (all major peripheral lymph nodes: inguinal, superficial cervical, axillary, brachial) and spleens of DO11.10 Ca−/− mice by complement-mediated lysis of CD8+ (clone 3.155), CD24+ (clone J11D), and MHC-II+ (clone M5/114) cells. Naïve CD4+ T cells were then sorted using a FACSAria (BD Biosciences) for collection of CD62L^hi^ CD44^lo^ cells. Cells were incubated with irradiated (2500 rads) APCs, prepared by complement-mediated lysis of Thy1.2+ cells from BALB/c mice, and primed with 1 µM OVA [323–339] peptide in the presence of 10 U/mL rhIL-2, 20 ng/mL IL-12, and 40 µg/mL α-IL-4 (clone 11B11) for 3d. Cells were split 1∶2 in 10 U/mL rhIL-2 on day 3 and harvested on day 5 for adoptive transfer. 1×10^6^ OVA-specific Th1 effector cells (Thy1.1+) were transferred i.v. into OVA/CFA immunized (12 d prior) BALB/c mice (Thy1.2+).

### Expression analysis of ear homogenates

BALB/c mice were immunized as previously indicated. Whole ears were removed from anesthetized animals and homogenized in 1 mL of Trizol (Life Technologies). RNA was isolated following the manufacturer's instructions and RNA quality was assessed using the Agilent 2100 Bioanalyzer (Agilent Technologies). 1200 ng RNA was converted to cDNA using Life Technologie's High Capacity cDNA Reverse Transcription kit. 500 ng cDNA in a volume of 100 ul was loaded per port onto customized 384-well microfluidic (TaqMan Low Density Array or TLDA) cards (Applied Biosystems (ABI) by Life Technologies). Four samples were loaded per TLDA card and run on an ABI 7900HT system as per manufacturer's recommendations. Data was analyzed using SDS v2.3 software. Delta Ct was generated by normalizing the Ct values for each gene to HPRT. Changes in gene expression were determined relative to PBS injected ears (for OVA/CFA experiments) or a single PBS control sample as a reference mouse (for *L. major* experiments). Genes were selected based on treatment p-values from 2-way ANOVAs (treatment and experiment day) using Prism (Graphpad Software). Matlab (The Mathworks) was used for gene clustering and for creating the heat map.

## Results

### Increased Th1 chemokine gene expression following short-term IL-4 blockade

**Figure 1:**
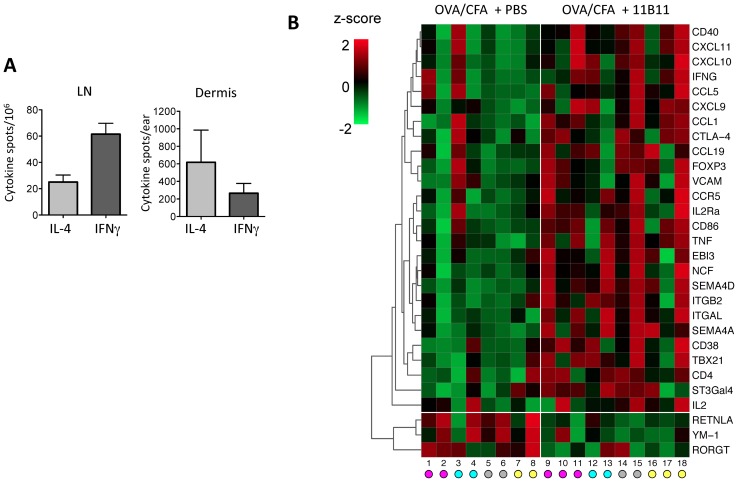
Short-term IL-4 blockade modulates the inflamed tissue environment. **A**) Antigen-specific IL-4 and IFNγ production by ELISPOT, day 7 following OVA/CFA immunization. **B**) Heat map of TLDA array genes (n = 94 genes examined, Table S1) expressed in the ear dermis of control and anti-IL-4 treated OVA/CFA immunized mice. Anti-IL-4 treatment (or PBS) was administered on day 7 and 10 post-immunization and RNA extracted on day 14. 29 genes (rows) were identified based on the treatment p-value (<0.05) after a 2-way ANOVA. The columns correspond to 18 samples. The colored circles beneath the heat map indicate which of the four experiments the sample was processed in. Color in the heat map corresponds to the z-score of relative expression data. For each gene, the z-score was computed separately in each of the four experiments rather than using all 18 samples together. Genes were clustered using correlation and average linkage.

**Figure 2:**
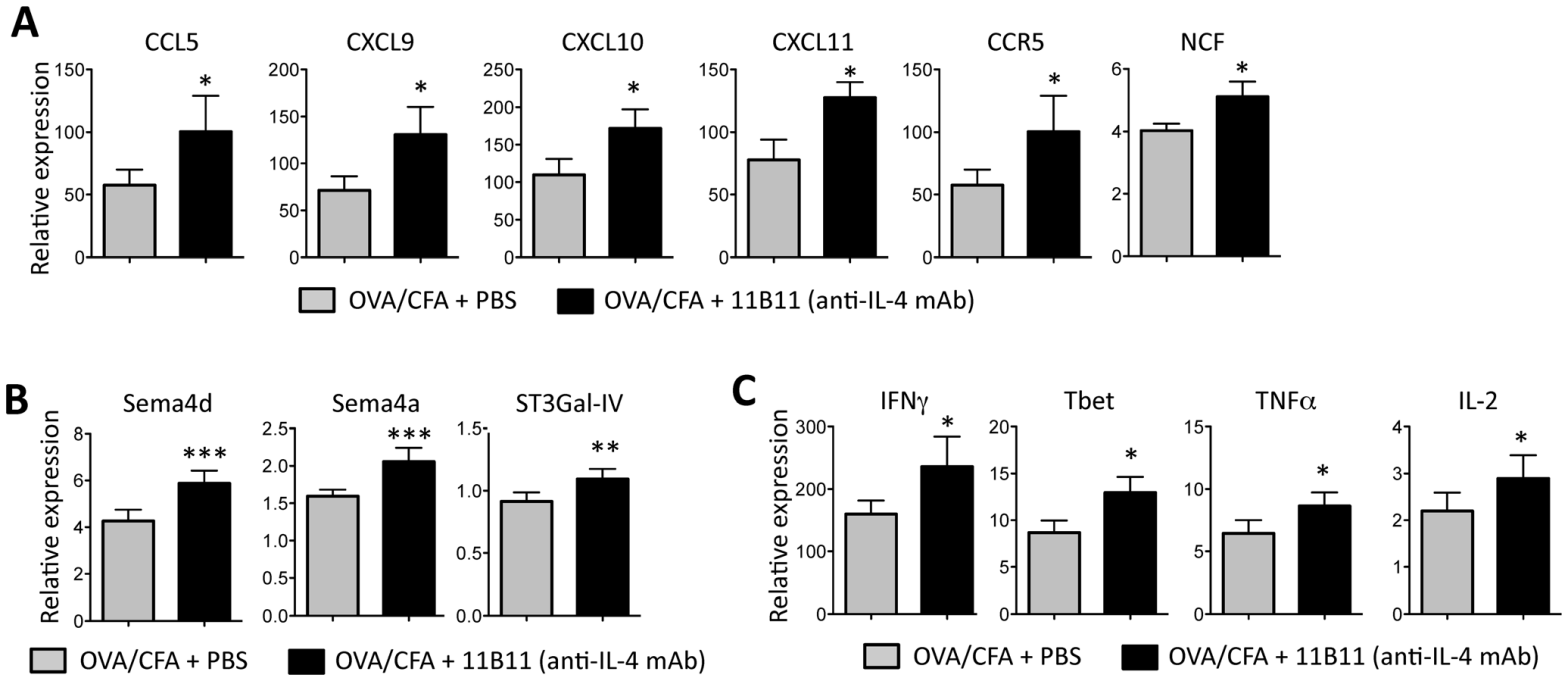
Immune trafficking genes upregulated following short-term IL-4 blockade. **A–C**) Genes expressed in the ear dermis of control and anti-IL-4 treated OVA/CFA immunized mice. Anti-IL-4 treatment (or PBS) was administered on day 7 and 10 post-OVA/CFA immunization and RNA extracted on day 14. Relative gene expression for those genes significantly different between control and anti-IL-4 treated groups. Statistics by two-way ANOVA: *p<0.05, **p<0.01, ***p<0.001. Data from 4 independent experiments.

In order to define the role of IL-4 in inflamed tissues we first analyzed the changes in gene expression in the CFA/OVA immunized ear dermis following short-term IL-4 blockade using the anti-IL-4 mAb 11B11. CFA/OVA immunization results in a mixed effector T cell response containing both IL-4 and IFNγ producing CD4+ T cells in the lymph node (LN) and immunized dermis (Fig. 1A). To isolate the role of IL-4 in local dermal gene expression from its role in the generation of IL-4 producing Th2 cells in the LN, we immunized mice with CFA/OVA and established robust effector T cell responses in the draining LN (dLN) and in the inflamed dermis prior to IL-4 blockade. Cytokine production in the draining lymph node peaked 7–10 days post-immunization (data not shown). Anti-IL-4 mAb (1 mg per mouse per treatment or PBS control) was administered i.p. on day 7 and day 10 and the inflamed dermis harvested on day 14 for mRNA isolation. Given the wide array of both hematopoietic and non-hematopoietic cells types that could be receptive to IL-4 signals, whole ear mRNA was analyzed to obtain a broad unbiased pattern of changes in gene expression. Using a custom 96-gene array micro-fluidics card we screened for changes in expression of 94 inflammatory genes (for gene list see Table S1). 29 genes were identified using 2-way ANOVAs that were significantly differentially expressed between the two groups and their clustered expression patterns across all 18 samples are shown in a heat map to aid in visualization (Fig. 1B). The magnitude of gene expression changes was variable between experiments and within groups, probably reflecting biological variability in inflammation at the immunization site and variability in IL-4-blockade in individual mice. Nonetheless, analysis of the whole tissue revealed small but significant fold changes in gene expression between anti-IL-4 and control groups. The majority of changes were genes that were upregulated following IL-4 neutralization (26 of 29 differentially expressed genes). Of those genes upregulated, over half of the genes were associated with lymphocyte trafficking and included semaphorins, chemokines and chemokine receptors (Fig. 1B, Fig. 2A, 2B). In particular, the increased chemokines were all Th1-associated chemokines, CXCR3-ligands and CCL5 (Fig. 2A). In agreement with an increase in Th1-chemokines, Th1 genes IFNγ, Tbet, IL-2 and TNFα were all significantly elevated in the dermis of anti-IL-4 treated mice (Fig. 2C). Thus, short-term depletion of IL-4 induces expression of genes associated with Th1 recruitment suggesting that IL-4 may play a role in limiting the accumulation of IFNγ-producing effector T cells in the inflamed dermal tissue.

**Figure 3:**
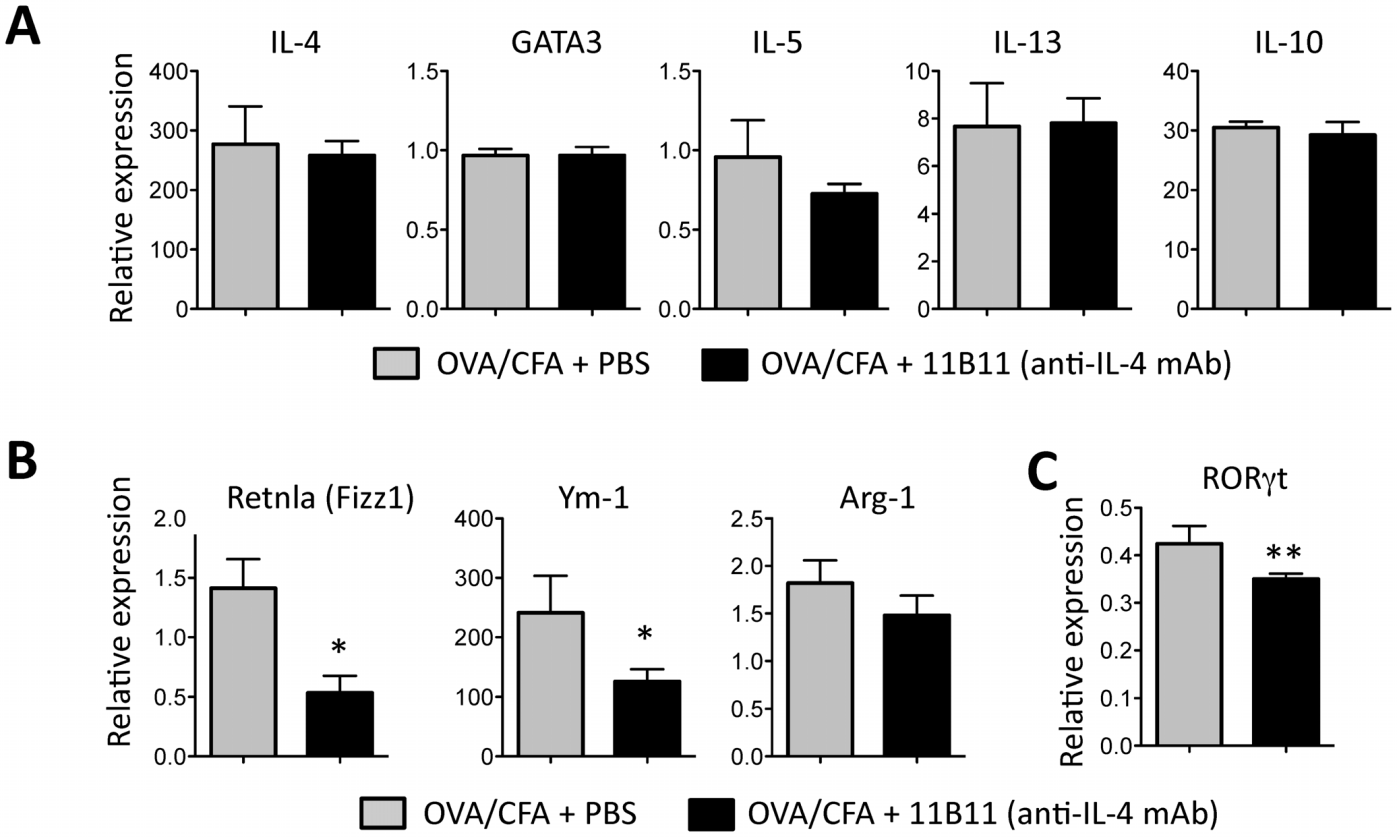
Alternatively activated macrophage genes down-regulated following short-term IL-4 blockade. **A–C**) Gene expression in the ear dermis of control and anti-IL-4 treated OVA/CFA immunized mice. Anti-IL-4 treatment was administered on day 7 and 10 post-immunization and RNA extracted on day 14. **A**) Relative gene expression for Th2-associated genes, all genes shown were not significantly differentially expressed between groups by two-way ANOVA. **B–C**) Genes significantly down-regulated between anti-IL-4 treated and control groups. Statistics by two-way ANOVA: *p<0.05, **p<0.01. Data from 4 independent experiments.

Surprisingly, only 3 genes were significantly reduced in the absence of IL-4 (Fig. 1B, Fig. 3B, 3C). IL-4 blockade did not lead to the reciprocal loss of Th2 cytokines (Fig. 3A), consistent with anti-IL-4 mAb treatment occurring after initial Th2 differentiation. Despite the increase in Th1-associated chemokines, we did not observe the reciprocal decrease in Type 2-associated chemokines, CCL1, CCL-7, CCL11, or CCR3 and CCR4 following IL-4 blockade (CCL17 and CCL22 were not examined) (data not shown). Rather, genes sensitive to short-term IL-4 blockade were those linked to an alternatively activated, or M2, macrophage phenotype (AAM). Expression of Retnla/Fizz1 and Ym-1 were both significantly reduced following IL-4 depletion (Fig. 1B, Fig. 3B). Arg-1 expression also clustered with genes down-regulated following IL-4 blockade (data not shown) but did not reach statistical significance (Fig. 3B). The change in the type or function of tissue macrophages, a loss of M2 genes and inferred gain of Th1-attracting chemokines, may facilitate the increase in Th1 gene expression. In addition, IL-4 blockade also led to a loss of RORγt expression (Fig. 3C) perhaps reflecting the link between IL-4/IL-9 and Th17s [35], [36], [37] however, in these short-term blocking studies we did not see a significant change in IL-17a or IL-23 gene expression.

### Short-term IL-4 blockade increases Th1 cell trafficking to the inflamed dermis

**Figure 4:**
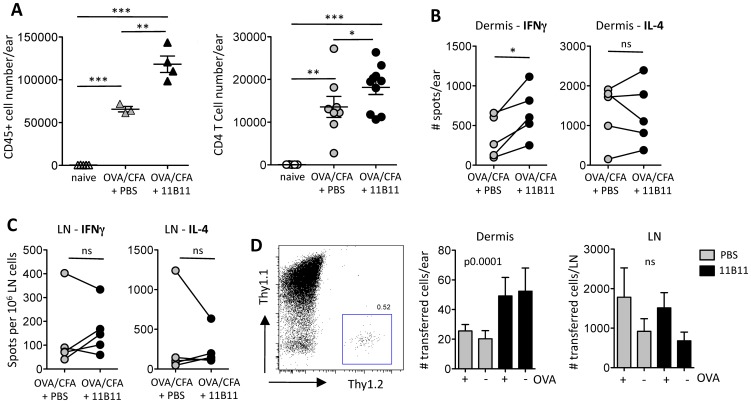
Increased T cell trafficking to inflamed dermis following short-term IL-4 blockade. **A**) Increased immune cell accumulation in the immunized dermis: left panel, number of CD45+ cells from one of 4 representative experiments, each symbol represents an individual mouse; right panel, number of CD4+CD3+ T cells, data from 3 experiments, each symbol represents an individual mouse. Statistics by two-tailed T test: *p<0.05, **p<0.01. **B**) Increased number of OVA-specific IFNγ producers in the ear dermis after IL-4 blockade, by ELISPOT. Each symbol represents an individual experiment with data obtained from pooled tissue from 3–4 mice; control and anti-IL-4 treated groups from the same experiment are paired. Statistics by paired T test, *p<0.05. **C**) cytokine production in draining LN from same experiments as in B). Statistics by paired T test, ns = p>0.05. **D**) Adoptive transfer of in vitro generated Th1 DO11.10+ Thy1.2+ T cells into Thy1.1+ mice immunized with OVA/CFA in one ear (+OVA) and CFA in other ear (−OVA). Mice were treated with anti-IL-4 (11B11) on days 7 and 10 after immunization, and Th1 cells transferred on day 12 after immunization. Left panel, representative FACS profile of endogenous (Thy1.1+) and transferred cells (Thy1.2+) in ear dermis 24 h after cell transfer. Middle and right panels, number of transferred cells in dermis or draining LN, respectively, 24 hours after cell transfer. Statistics by two-way ANOVA.

The gene expression data suggested that IL-4 may negatively regulate the recruitment of Th1 cells to the inflamed dermis. To determine cellular changes following short-term IL-4 blockade, we analyzed leukocyte number and function in the inflamed dermis and dLN. Blocking IL-4 led to a significant increase in the total number of leukocytes (CD45+) in the inflamed dermis including increases in CD4+ T cells (Fig. 4A). Examination of antigen-specific T cell function by ex vivo ELISPOT, revealed that IFNγ-producing T cells (but not IL-4 producers) were significantly increased in the dermis after IL-4 depletion (Fig. 4B) consistent with the local increase in Th1-chemokines. The effect appeared to be a local tissue-specific event as numbers of IFNγ and IL-4 producers in the dLN did not consistently change on IL-4 blockade (Fig. 4C). The increased accumulation of IFNγ producers could arise from recruitment of new effectors into the inflamed tissue or from better retention/survival of existing Th1 cells in the tissue. To directly test the ability of the IL-4-depleted environment to better recruit Th1 cells, we used an adoptive transfer approach with in vitro-primed OVA-specific Th1 cells (DO11.10+ TCR Tg+ T cells). Recipient mice (Thy1.1+) were immunized with OVA/CFA in the left ear and CFA alone in the right ear and treated on day 7 and day 10 post-immunization with anti-IL-4 mAb or PBS. On day 12 post-immunization, 1×10^6^ in vitro primed DO11.10+ Thy1.2+ Th1 cells were transferred i.v. into the mice and their initial accumulation in the inflamed ear and dLN assessed 24 h later (Fig. 4D). Small numbers of transferred OVA-specific DO11.10+ Thy1.2+ Th1 cells could be clearly identified in the inflamed dermis (Fig. 4D, left panel). Regardless of the presence of antigen (+/−OVA), more OVA-specific DO11.10 Thy1.2+ Th1 cells were found in the inflamed ear of recipients that had been treated with anti-IL-4 mAb than PBS (Fig. 4D), or isotype control Ab (data not shown). This was a local difference in OVA-specific Th1 accumulation in the inflamed tissue as there was no change in the number of OVA-specific Th1 cells in the dLN following IL-4 neutralization. Thus, short-term IL-4 blockade enhances antigen-specific Th1 trafficking to the inflamed dermal tissue.

### *Leishmania major*-infected Th2-rich dermal tissue displays a reciprocal pattern of altered gene expression

**Figure 5:**
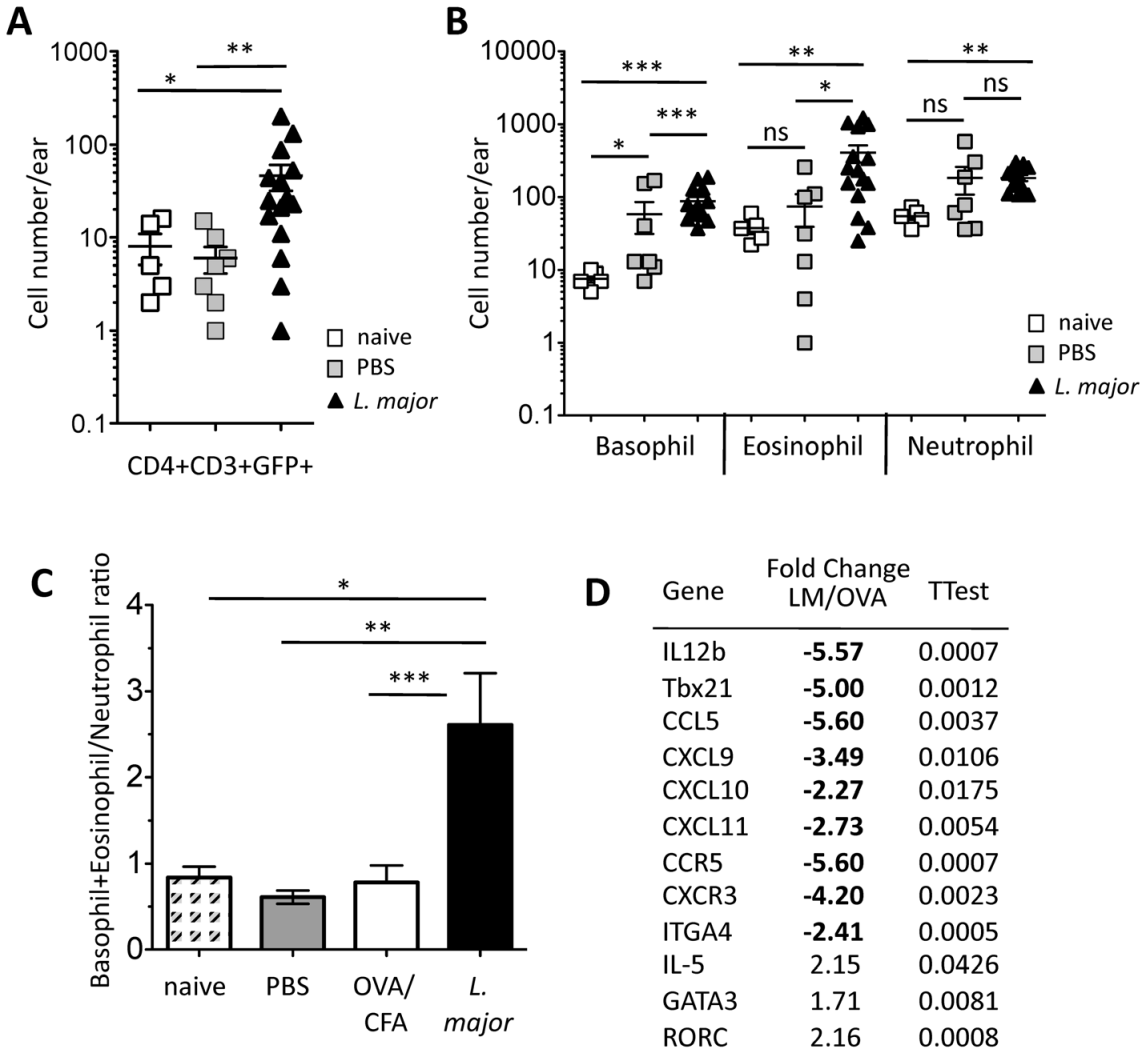
Early Type 2 immunity in the *Leishmania major* infected dermis with broad down-regulation of Type 1 chemokines. **A**) Number of CD4+CD3+GFP+ T cells in the *L. major* infected ear dermis 2 weeks post-infection compared to PBS-injected control and ears from naïve mice. **B**) Number of innate cell types in *L. major* infected ear 2 weeks post-infection compared to PBS-injected control and naïve mice. **C**) Basophil+Eosinophil/Neutrophil ratio of cells in the dermis 2 weeks post-infection compared to PBS or OVA/CFA immunization and naïve mice. **A–C**, Statistics by Mann Whitney: *p<0.05, **p<0.01, ***p<0.001. **D**) Fold change in gene expression in the ear dermis of *L. major*-infected versus OVA/CFA immunized. RNA at 2 weeks post-infection/immunization analyzed by TLDA gene array as in Table S1. Table shows all genes statistically (TTest) differentially expressed between groups: bold, those genes underrepresented in the *L. major* infected dermis; not bold, those genes enriched in *L. major* infected dermis compared to OVA/CFA. Data from 3 independent experiments.

To determine the biological significance of IL-4 effects on gene expression for Th1 trafficking we tested the effect of short-term IL-4 blockade in the mouse model of *Leishmania major* infection. Clearance of *L. major* is heavily dependent on the production of IFNγ by CD4+ T cells in the infected dermis. However, early studies revealed that IL-4 needed to be neutralized within the first few days of infection in order for long-term clearance of infection in susceptible mice [38] suggesting that, once established, the IL-4 response dominates immunity to *Leishmania*. Indeed, analysis of the *L. major* infected dermis using a multi-parameter FACS panel and infection of IL-4-GFP reporter mice (4get) [33] revealed GFP+ immune cells with IL-4 potential in both adaptive and innate compartments. *Leishmania major* infection of BALB/c mice in the ear dermis led to the early influx of IL-4/GFP+ CD4+ T cells (Fig. 5A) and a Type 2 biased innate infiltrate with an increase in both basophil and eosinophil numbers (but not neutrophil) compared to PBS injected controls (Fig. 5B, 5C) [39]. Using the same gene expression array as for the OVA/CFA experiments, we asked if this IL-4-dominated microenvironment generated with *L. major* infection led to the reverse pattern of gene expression from that seen with IL-4 blockade. mRNA was isolated from the 2 week (IL-4 dominated) infected *L. major* dermis and compared to the (IL-4/IFNγ mixed) OVA/CFA dermis. We found a surprisingly similar, but opposing, pattern of gene regulation in the *L. major* infected dermis (Fig. 5D) compared to our IL-4 blocking studies (Fig. 1B, 2A). The same genes associated with Th1 trafficking that were upregulated on IL-4 neutralization (Fig. 1B, 2A) were strikingly down regulated in the IL-4-rich *L. major* infected dermis, extending the range of gene expression changes we originally described [28]. Of note, very few genes were upregulated in the *L.major* dermis compared to OVA/CFA but, as with IL-4 blockade, RORγt positively associated with IL-4 (Fig. 5D) consistent with elevated IL-17 responses in *L. major* infected susceptible BALB/c mice [40], although at the timepoints in this study we saw no increases in gene expression for IL-17a or IL-23.

### IL-4 blockade increases T cell infiltration into the *L. major* infected dermis and improves parasite clearance

**Figure 6:**
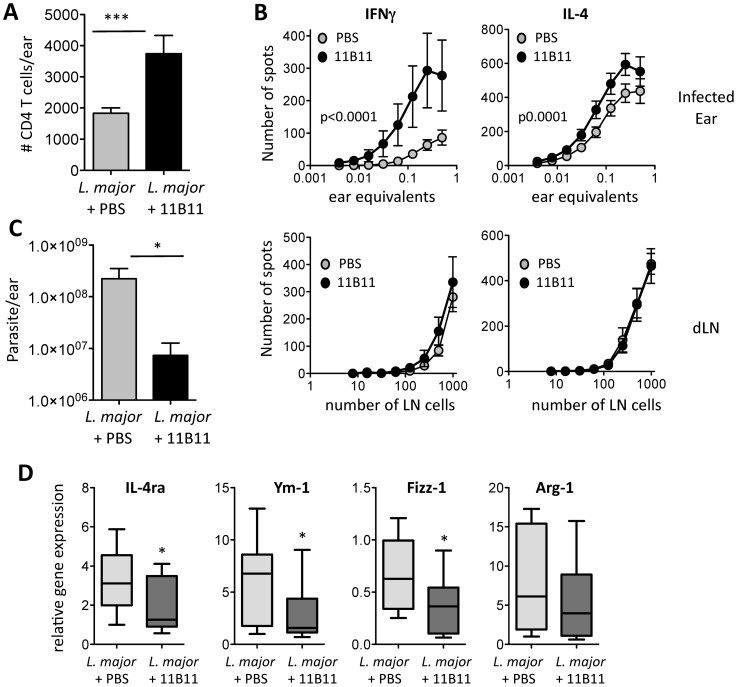
Increased T cell infiltration and improved *L. major* parasite clearance following short-term IL-4 blockade. **A**) Increased immune cell accumulation after anti-IL-4 treatment in the *L. major* infected dermis: number of CD4+CD3+ T cells, representative data from one of three experiments. Anti-IL-4 treatment was administered on day 7 and 10 post-infection and cells analyzed on day 14. **B**) Anti-*Leishmania* IFNγ and IL-4 producing T cells by ELISPOT in infected ear (top) and draining LN (bottom) after anti-IL-4 treatment or PBS as in A). Data from 4 independent experiments. Statistics comparing PBS and 11B11 by ANOVA. **C**) Parasite load in ear dermis after *L. major* infection and anti-IL-4 treatment on day 7 and day 10, infected tissue harvested on day 21. A) and C) Statistics by Mann Whitney: *p<0.05. ***p<0.001. **D**) Relative gene expression in the *L. major*-infected dermis after 11B11 or PBS treatment (as in A); 8 mice per group, from two independent experiments. Statistics by one-tailed T test, * <0.05.

*Leishmania major* infected BALB/c mice were treated with 11B11 or PBS (or isotype control, data not shown) on days 7 and 10 post-infection and mice sacrificed on day 14 to analyze immune cell numbers and function and on day 21 to determine the effect on pathogen clearance. Similar to the OVA/CFA immunization, IL-4 blockade led to a significant increase in total leukocyte numbers in the infected tissue including elevated CD4+ T cells numbers (Fig. 6A), with no change in CD4+ T cell dLN numbers (data not shown). Similar increases in CD4+ T cell number in the infected dermis after IL-4 blockade were seen in C57BL/6 mice (2 week *L. major* infected, data not shown). Functionally, anti-IL-4 treatment led to the selective enrichment of *Leishmania*-specific IFNγ-producers in the infected lesion (Fig. 6B). In line with these increases in T cell number and function, parasite numbers (determined by limiting dilution) were significantly attenuated with IL-4 blockade. Therefore, short-term IL-4 blockade leads to local immune changes that boost Th1 recruitment, enhance IFNγ production and result in improved pathogen clearance. Associated with this change in the cytokine micro-environment in the infected ear we found that gene expression for a number of genes linked to alternatively activated macrophages were significantly downregulated following transient IL-4 blockade (Fig. 6D). Thus, blocking IL-4 may enhance pathogen clearance in two ways: by increased IFNγ-dependent activation of leishmaniacidal macrophage function and reversal of the alternatively activated macrophage phenotype.

## Discussion

Cytokine production in the lymph node is well documented to regulate the type of effector T cells (Th1, Th2, Th17, iTreg) generated upon antigen challenge. Cytokines during Th differentiation play a role in promoting specific effector functions and antagonizing the generation of alternative effector subsets. These effects are due in part to STAT-mediated signals downstream of the cytokine receptors that control the expression of lineage-defining transcription factors and subsequent chromatin remodeling events that create permissive epigenetic modifications at certain gene loci while repressively marking gene loci for alternative effector functions. Although the stability of this differentiation process has been questioned in recent years [41], [42], [43], the result of many infectious challenges is the polarization of the immune response toward a dominant Th1, Th2 or Th17 functional T cell profile with the recruitment of their corresponding distinct innate cell types. In addition to induction of functionally distinct effector T cells, there is evidence to suggest that the polarizing cytokines are also required to expand or maintain a functionally distinct effector/memory response: the absence of IL-4/STAT6 signaling attenuates Th2 effector and memory responses [23], [44] while IL-12 can help to maintain Th1 function [45], [46]. A third role for IL-4 in sustaining a polarized Th2 response comes from studies that highlight the STAT6-dependence for the recruitment of Th2 and Type 2 innate effectors to sites of inflammation [21], [23], [24]. Indeed, IL-4 is known to promote the expression of Type 2-associated chemokines and chemokine receptors [20]. Our studies reveal an additional role for IL-4 in the negative regulation of chemokine expression associated with Th1 recruitment to inflamed/infected tissues. Short-term blockade of IL-4 led to increases in gene expression for a variety of chemokines in the inflamed dermal tissue including Th1-associated chemokines, CXCL9, 10 and 11, the Th1-associated semaphorin 4A and neutrophil chemotactic factor, NCF. The changes in gene expression correlated with increased accumulation of lymphocytes in the inflamed dermis and a selective increase in IFNγ-producers. Using *Leishmania major* infection to test the biological significance of changes in IL-4, we found that blocking IL-4 during infection also led to an increased number of IFNγ-producers in the infected dermis and improved pathogen control. A similar negative regulatory role for IL-4 in Th1 recruitment to sites of inflammation was observed in a model of Th1-driven pleuritis where administration of IL-4 significantly blocked cell trafficking to the pleural cavity [47].

Although IL-4 was neutralized systemically, the functional effects were observed in the inflamed/infected dermal tissue and not in the draining LN, probably reflecting the timing of anti-IL-4 treatment given after initial Th differentiation. The effect of IL-4 within the local inflamed microenvironment is likely to be multi-factorial. There have been a number of studies that have defined the STAT-6 requirements for the development of Type 2 inflammation where IL-4 signals appear necessary for the recruitment of Th2 cells and eosinophils to the lung during *Nippostrongylus brasiliensis* infection and for Th2 accumulation in the lung in allergic inflammation [3]. Roles for STAT-6 signaling in non-hematopoietic cells in tissues and in a non-T cell hematopoietic cell type have been implicated [24], [25], [48] but the specific cellular players remain to be identified. STAT-6 inducible chemokine expression in tissues and possible STAT-6 dependent changes in endothelial adhesion molecules [49], [50], [51], [52] are thought to contribute to increased Type 2 immune cell recruitment and could also be targets for the negative regulation of immune function by IL-4.

In association with increases in Th1 related genes, Foxp3 expression was also enhanced following anti-IL-4 treatment (Fig. 1B). Additional experiments to analyze the accumulation of Foxp3+ Tregs in the tissue have suggested the change in Foxp3 gene expression reflects a small, but not significant, increase in the frequency of Foxp3+ Tregs within the CD4 population in the inflamed dermis after IL-4 blockade (data not shown). Thus, the increase in Foxp3 gene expression likely reflects the general increase in lymphocyte numbers in the ear after IL-4 blockade, rather than a specific effect of IL-4 on Treg accumulation. Future studies on the phenotype and function of these Tregs would be informative, given reports that a Th1 environment can promote the recruitment of Foxp3+Tbet+ Tregs to sites of inflammation [53], [54].

The mechanisms underlying IL-4 signals that negatively regulate Th1 immune recruitment require further investigation. Our data support a role for IL-4 in the negative regulation of Type 1 chemokines. During Type 1 inflammation many chemokines are upregulated in stromal and immune cell types in the infected tissue and our studies have not identified the source of the upregulated chemokines. In Type 2 inflammation, an as yet unidentified tissue-resident dendritic or macrophage population has been implicated in regulating leukocyte recruitment [24]. In combination with the loss of AAM markers Fizz1 and Ym-1 following IL-4 blockade, one interesting possibility is that IL-4 controls the balance of chemokine expression in the tissue microenvironment by modulating macrophage functions [2], [17]. IL-4 and IL-13 play key roles in directing the induction of the AAM phenotype [16], [55] that is associated with increased expression of AAM signature genes (*Arg1, Fizz1, Ym1*) and a down-regulation of pro-inflammatory macrophage functions such as NO production [17]. Interestingly, the macrophage phenotype appears flexible, with macrophages rapidly switching between functional phenotypes (classical or alternatively activated) depending on signals from the microenvironment [56]. Indeed, genes associated with AAM were significantly downregulated in the *L.major* infected dermis following short-term IL-4 blockade. Thus, transient blockade of IL-4 may reverse the tissue resident macrophage phenotype and contribute to the local promotion of Th1-associated chemokine production.

There have been a number of studies that implicate negative regulatory roles for IL-4 and leukocyte trafficking. IL-4 can down-regulate VLA-4 expression required for CD8 and Th1 cell trafficking into tumors [57], [58] and can down-regulate CCR6 on immature dendritic cells possibly limiting their trafficking into inflamed peripheral tissues [59]. In vitro studies have also suggested that Th2 cytokines including IL-4 can block T cell chemotaxis to chemokines such as IL-8 and RANTES [60] and inhibit monocyte expression of IL-8 [61]. In addition, IL-4 could modify the expression of numerous molecules on the vascular endothelium [51], [52], although studies have mainly focused on the positive regulation of endothelial expression of chemokines (eotaxin) and adhesion molecules (VCAM-1) for Type 2 infiltration [49]. Therefore it remains possible that the initiating factor following IL-4 blockade is the recruitment of Th1 cells that, themselves, then promote the increase in chemokine expression we observed. Indeed, a positive feedback loop between cytokine and chemokine production amplifies Th1 recruitment [20], [22], [26] with IFNγ-activated STAT1 inducing upregulation of CXCR3 ligands in tissue resident cells [27].

More recently, the Voehringer group have highlighted an interesting STAT-6-dependent negative regulation of effector cell accumulation in the lungs of Nippostrongylus brasiliensis infected mice [62]. Similar to our studies with short-term IL-4 blockade, the total number of CD4 T cells in the lungs of infected mice was elevated in the absence of STAT-6 signaling and STAT-6 deficient T cells preferentially accumulated in the lung in competitive reconstitution experiments with WT and STAT-6−/− T cells. Interestingly, the study concluded that the absence of IL-4/IL-13 signals led to improved survival of effector T cells in infected tissues. Our adoptive transfers of Th1 effectors clearly show an early increase in tissue recruitment following IL-4 blockade but the net accumulation of cells in the inflamed tissue may also be controlled at the level of increased survival or expansion. In a parallel study, IFNγ was shown to limit Th1 cell adhesion to inflamed endothelium [63] and blockade of IFNγ signaling elevated accumulation of leukocytes into the skin during contact hypersensitivity. Thus, in contrast to the classic positive feedback loops for polarized effector T cell recruitment, effector cytokines may also attenuate the recruitment of other T cells of the same effector phenotype [64]. Such regulation of lymphocyte accumulation in inflamed tissues by recruitment and or expansion may serve to limit immune pathology.

In the context of infection, many pathogens induce IL-4 that serves to ‘deviate’ immunity away from an anti-microbial Th1 response. This is perhaps best highlighted in TB where IL-4 severely attenuates protective immunity to *M. tuberculosis* [30], [65]. Our data suggest that one of the consequences of elevated IL-4 in the chronically infected microenvironment is to limit the expression of Th1-assoicated chemokines and reduce Th1 accumulation in the infected tissue; ultimately prolonging pathogen residency in the host. As supported by our studies with *Leishmania major* infection, the ability to recruit anti-microbial Th1 cells can be promoted by transiently blocking IL-4. Similarly, dramatic effects are seen on tumor control following IL-4 ablation [66]. Thus, for many chronic infections (and tumors [18]), the central (LN) generation of protective Th1 immunity may be sufficient to control infection but local IL-4-rich tissue microenvironments prevent their recruitment and limit anti-microbial function [30]. This may be particularly destructive in the setting of co-infection [30], [32], [67], [68], [69], [70], [71]. Understanding the molecular events that are negatively regulated by IL-4 will be essential for the design of novel therapeutics that target chronic infection by inhibiting IL-4's control of immunity in tissues.

## Supporting Information

Table S1 **List of 96 genes (93 genes of interest plus 3 endogenous controls) included in the custom-designed microfluidics card.** Taqman Assay ID for each gene is listed to the right of the gene symbol.
(DOCX)
