# Supplementary material for: IL-4 Attenuates Th1-Associated Chemokine Expression and Th1 Trafficking to Inflamed Tissues and Limits Pathogen Clearance
Source: PLoS One. 2013 Aug 26;8(8):e71949. doi: 10.1371/journal.pone.0071949 (PMC3753298; doi:10.1371/journal.pone.0071949)
Supplement: Table S1 — List of 96 genes (93 genes of interest plus 3 endogenous controls) included in the custom-designed microfluidics card. Taqman Assay ID for each gene is listed to the right of the gene symbol. (DOCX) [file pone.0071949.s001.docx]

| **Gene Symbol** | **Assay ID** | **Gene Symbol** | **Assay ID** | **Gene Symbol** | **Assay ID** |
| --- | --- | --- | --- | --- | --- |
| 18S | Hs99999901_s1 | Csf2 | Mm00438328_m1 | Il3 | Mm00439631_m1 |
| Arg1 | Mm01190441_g1 | Ctla4 | Mm00486849_m1 | Il4 | Mm00445259_m1 |
| Bax | Mm00432050_m1 | Cxcl10 | Mm00445235_m1 | Il5 | Mm00439646_m1 |
| Bcl2 | Mm00477631_m1 | Cxcl11 | Mm00444662_m1 | Il6 | Mm00446190_m1 |
| Bcl2l1 | Mm00437783_m1 | Cxcl9 | Mm00434946_m1 | Il7 | Mm00434291_m1 |
| Ccl1 | Mm00441236_m1 | Cxcr3 | Mm00438259_m1 | Il9 | Mm00434305_m1 |
| Ccl11 | Mm00441238_m1 | Ebi3 | Mm00469294_m1 | Itga4 | Mm00439770_m1 |
| Ccl19 | Mm00839967_g1 | Epx | Mm00514768_m1 | Itgal | Mm00801807_m1 |
| Ccl2 | Mm00441242_m1 | Fas | Mm00433237_m1 | Itgb1 | Mm01253227_m1 |
| Ccl3 | Mm00441258_m1 | Fasl | Mm00438864_m1 | Itgb2 | Mm00434523_g1 |
| Ccl5 | Mm01302428_m1 | Foxp3 | Mm00475156_m1 | Lta | Mm00440227_m1 |
| Ccl7 | Mm00443113_m1 | Fut7 | Mm01330673_g1 | Ncf1 | Mm00447921_m1 |
| Ccr2 | Mm99999051_gH | Gapdh | Mm99999915_g1 | Nos2 | Mm00440485_m1 |
| Ccr3 | Mm01216172_m1 | Gata3 | Mm00484683_m1 | Plxna1 | Mm00501110_m1 |
| Ccr4 | Mm00438271_m1 | Gcnt1 | Mm02010556_s1 | Prf1 | Mm00812512_m1 |
| Ccr5 | Mm01216171_m1 | Gzmb | Mm00442834_m1 | Retnla | Mm00445109_m1 |
| Ccr7 | Mm00432608_m1 | Hprt1 | Mm00446968_m1 | Rorc | Mm01261022_m1 |
| Cd19 | Mm00515420_m1 | Icos | Mm00497600_m1 | Sele | Mm00441278_m1 |
| Cd28 | Mm00483137_m1 | Ifng | Mm00801778_m1 | Selp | Mm00441295_m1 |
| Cd34 | Mm00519283_m1 | Il10 | Mm00439616_m1 | Sema3a | Mm00436469_m1 |
| Cd38 | Mm00483146_m1 | Il12a | Mm00434165_m1 | Sema4a | Mm00443140_m1 |
| Cd3e | Mm00599683_m1 | Il12b | Mm01288992_m1 | Sema4d | Mm00443147_m1 |
| Cd4 | Mm00442754_m1 | Il13 | Mm00434204_m1 | Sema6d | Mm00553142_m1 |
| Cd40 | Mm00441895_m1 | Il15 | Mm00434210_m1 | Sema7a | Mm00441361_m1 |
| Cd40lg | Mm00441911_m1 | Il17a | Mm00439619_m1 | Smad3 | Mm00489637_m1 |
| Cd68 | Mm00839636_g1 | Il18 | Mm00434225_m1 | Smad7 | Mm00484741_m1 |
| Cd80 | Mm00711660_m1 | Il1a | Mm00439620_m1 | St3gal4 | Mm00501503_m1 |
| Cd86 | Mm00444543_m1 | Il1b | Mm00434228_m1 | Tbx21 | Mm00450960_m1 |
| Cd8a | Mm01182107_g1 | Il2 | Mm00434256_m1 | Tgfb1 | Mm00441724_m1 |
| Chi3l3 | Mm00657889_mH | Il22;Iltifb | Mm00444241_m1 | Timd2 | Mm00506693_m1 |
| Cma1 | Mm00487638_m1 | Il23a | Mm00518984_m1 | Tnf | Mm00443258_m1 |
| Csf1 | Mm00432688_m1 | Il2ra | Mm00434261_m1 | Vcam1 | Mm00449197_m1 |

**Supplemental Table 1**

List of 96 genes (93 genes of interest plus 3 endogenous controls) included in the custom-designed microfluidics card. Taqman Assay ID for each gene is listed to the right of the gene symbol.
